# The gut-microbiota-brain axis in a Spanish population in the aftermath of the COVID-19 pandemic: microbiota composition linked to anxiety, trauma, and depression profiles

**DOI:** 10.1080/19490976.2022.2162306

**Published:** 2023-01-18

**Authors:** Stefanie Malan-Müller, Mireia Valles-Colomer, Tomás Palomo, Juan C. Leza

**Affiliations:** a Department of Pharmacology and Toxicology, Faculty of Medicine, University Complutense Madrid (UCM), Madrid, Spain; b Biomedical Network Research Center of Mental Health (CIBERSAM), Institute of Health Carlos III, Madrid, Spain; c Neurochemistry Research Institute UCM, Hospital 12 de Octubre Research Institute (Imas12), Madrid, Spain; d Department of Cellular Computational and Integrative Biology, University of Trento, Trento, Italy

**Keywords:** Microbiome, gut-microbiota-brain axis, mental health, anxiety, depression, trauma, COVID-19, posttraumatic stress disorder

## Abstract

The prevalence of anxiety and depression soared following the COVID-19 pandemic. To effectively treat these conditions, a comprehensive understanding of all etiological factors is needed. This study investigated fecal microbial features associated with mental health outcomes (symptoms of anxiety, depression, or posttraumatic stress disorder (PTSD)) in a Spanish cohort in the aftermath of the COVID-19 pandemic. Microbial communities from stool samples were profiled in 198 individuals who completed validated, self-report questionnaires. 16S ribosomal RNA gene V3-4 amplicon sequencing was performed. Microbial diversity and community structure were analyzed, together with relative taxonomic abundance. In our cohort of N=198, 17.17% reported depressive symptoms, 37.37% state anxiety symptoms, 40.90% trait anxiety symptoms, and 8.08% PTSD symptoms, with high levels of comorbidity. Individuals with trait anxiety had lower Simpson’s diversity. *Fusicatenibacter saccharivorans* was reduced in individuals with comorbid PTSD + depression + state and trait anxiety symptoms, whilst an expansion of Proteobacteria and depletion of Synergistetes phyla were noted in individuals with depressive symptoms. The relative abundance of *Anaerostipes* was positively correlated with childhood trauma, and higher levels of *Turicibacter sanguinis* and lower levels of Lentisphaerae were found in individuals who experienced life-threatening traumas. COVID-19 infection and vaccination influenced the overall microbial composition and were associated with distinct relative taxonomic abundance profiles. These findings will help lay the foundation for future studies to identify microbial role players in symptoms of anxiety, depression, and PTSD and provide future therapeutic targets to improve mental health outcomes.

## Introduction

Depression and anxiety disorders are among the most prevalent neuropsychiatric disorders, with an estimated 322 million people living with depression and 264 million living with an anxiety disorder (including PTSD).^1^ PTSD, currently classified as a trauma- and stress-related disorder, can develop following exposure to a potentially traumatic event.^2^ The global prevalence of stress, anxiety, depression, and PTSD soared during the COVID-19 pandemic.^3,4^ The burden of these diseases is further compounded by non-response and non-adherence to the available treatments.^5,6^ Many patients with depression experience relapse,^7^ and each successive episode is more severe and increases resistance to treatment.^8^ More than one-third of patients with major depressive disorder (MDD) have an inadequate or partial response to initial treatment.^9^ Adherence to psychiatric treatment is further hindered by the long period until the onset of a clear clinical effect as well as the side-effect profiles of the medication.^5^ Furthermore, drug development for psychiatric conditions has been sluggish. These limitations in the treatment of neuropsychiatric disorders highlight the need to identify all role players in these complex conditions, to discover novel therapeutic targets to lighten the burden of disease.

The microbes we harbor play a vital part in health and disease.^10^ The gut-microbiota-brain axis describes the complex, tridirectional communication system between the gut, its microbiota, and the central nervous system;^11^ the gut microbiota can influence central nervous system functioning and behavior, whereas, stress and emotions can elicit effects on the microbiota. The tridirectional interactions within this axis modulate neural, hormonal, and immune responses,^12^ as well as intestinal and blood–brain barrier integrity.^13^ The composition of the gut microbiome is amenable to change, and several factors can alter its composition, including age,^14^ diet,^15^ exercise,^16^ the environment,^17^ cohabitation (more so than genetic relatedness), ^17^ medication use,^17^ disease,^18,19^ childhood living conditions and exposures,^17^ and traumatic life events.^20^

Clinical data on the gut microbiome in mental health disorders are dominated by studies focused on depressive cohorts, whilst data for anxiety and stress-related disorders are somewhat limited. The systematic reviews by Sanada et al., ^21^ and Simpson et al., ^22^ provide detailed information on gut microbiome findings in anxious and depressed cohorts and highlight consistent, but also conflicting results. Consolidation of findings is hampered by differences in methodology (including diagnostic tools, sample collection and preservation methods, sequencing methodologies, reference databases, and analysis approaches), small sample sizes, and various confounding factors (psychiatric and other prescription medication use, stool consistency, and diet), ^23,24^ which many studies do not incorporate into the analyses.

This study aimed to contribute to the current body of evidence by investigating the fecal microbiome in a naturalistic Spanish cohort of individuals with symptoms of anxiety, depression, and PTSD in the context of the recent COVID-19 pandemic as well as previous traumatic experiences.

## Results

### Clinical and demographic characteristics

**Figure 1.**
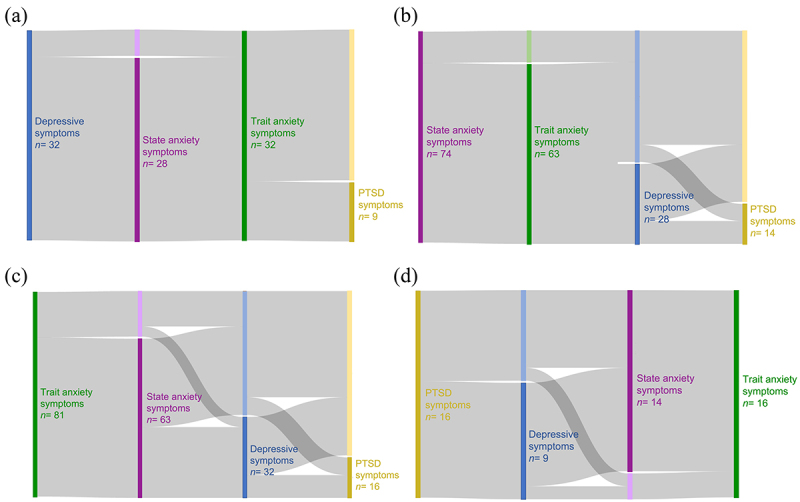
Sankey diagrams to illustrate the comorbid states in (a) the depressive symptom cohort, (b) state anxiety symptom cohort, (c) trait anxiety symptom cohort and (d) the PTSD symptom cohort.

In the total cohort of 198 individuals, 92 suffered from at least one or a combination of the psychiatric symptoms we assessed (depression, state, and trait anxiety, and PTSD), and 106 were mentally healthy controls, henceforth referred to as healthy controls (individuals who did not meet the cutoff criteria described for depression, state and trait anxiety, and PTSD). Of the 92 individuals with psychiatric symptoms, 32 presented with depressive symptoms (based on the Center for Epidemiologic Studies Depression [CESD] scores), 74 with state anxiety symptoms, 81 with trait anxiety symptoms (based on state-trait anxiety and depression inventory [STAI] scores), and 16 with symptoms of PTSD (based on the PTSD Checklist for DSM-5 with Life Events Checklist for the Diagnostic and Statistical Manual of Mental Disorders, Fifth Edition [PCL-5] scores). As expected, comorbidity was common with psychiatric symptoms;^25^ of the 63 individuals who had both state and trait anxiety symptoms, 28 also had depressive symptoms, 14 had comorbid PTSD symptoms and eight had PTSD and depressive symptoms. Of the 32 individuals who had depressive symptoms, all had trait anxiety symptoms, while 16 also had symptoms of PTSD (Figure 1a-d illustrate the comorbidities for each symptom cohort).

For the demographic and clinical data and subsequent analysis, specific sub-groups were defined. *Symptom cohorts* consisted of participants who met the cutoff criteria for the self-report questionnaires that evaluated depressive, state anxiety, trait anxiety, and PTSD symptoms, therefore four symptom cohorts. *Symptom control cohorts* consisted of participants with symptom scores *below* the cutoff criteria for each of the *separate* outcome measures, therefore, depression controls [CESD score ≤15, irrespective of the other scores], state anxiety controls [STAI-S scores < 41, irrespective of the other scores], trait anxiety controls [STAI-T scores < 45, irrespective of the other scores], and PTSD controls [PCL-5 score < 33, irrespective of the other scores]). The *healthy control cohort* consisted of participants with good mental health, who did not meet the cutoff criteria described for *any* of the outcome measures, namely depression, state and trait anxiety, and PTSD (CESD, STAI-S, STAI-T, and PCL-5 scores all below the cutoff). (Tables 1-4).

**Table 1.** Clinical and demographic characteristics of the study participants with depressive symptoms, compared to those without depressive symptoms and healthy controls.

	Total cohort (n = 198)			Depression (n = 32)			Depression controls (n = 166)			Healthy controls (n = 106)			Depression vs depression controls	Depression vs healthy controls
	mean ±SD			mean ±SD			mean ±SD			mean ±SD				
	*median*	*(IQR)*		*median*	*(IQR)*		*median*	*(IQR)*		*median*	*(IQR)*			
	*n (%)*			*n (%)*			*n (%)*			*n (%)*			p-value	p-value
Age, years	*37*	*(30– 43)*		*36*	*(32– 42)*		*37.5*	*(30– 43)*		*38*	*(30– 44)*		NS	NS
Female	139 (70%)			23 (72%)			116 (70%)			71 (67%)			NS	NS
Weight, kg	*65*	*(56– 73)*		66.03 ±13			*65*	*(57– 73)*		*65*	*(57– 73)*		NS	NS
BMI, kg/m^2^	*22.88*	*(21– 25)*		22.92 ±3			*22.92*	*(21– 25)*		*23.02*	*(21– 25)*		NS	NS
Autoimmune disease ever	24 (12%)			1 (3%)			23 (14%)			14 (13%)			NS	NS
IBD IB celiacs ever	33 (17%)			3 (9%)			30 (18%)			21 (20%)			NS	NS
Periodontitis ever	44 (22%)			7 (22%)			37 (22%)			22 (21%)			NS	NS
Psych meds ever	58 (29%)			17 (53%)			41 (25%)			21 (20%)			**< 0.005**	**< 0.001**
CTQ total	*31*	*(27– 39)*		*35*	*(31– 56)*		*30*	*(27– 39)*		*29*	*(27– 34)*		**< 0.005**	**< 0.001**
*WHOQOL*														
DOM1	*15.43*	*(14– 17)*		12.8 ±3			*16*	*(14– 17)*		*16.57*	*(15– 18)*		**< 0.001**	**< 0.001**
DOM2	*14.00*	*(12– 16)*		11.04 ±2			*14.67*	*(13– 16)*		15.55 ±2			**< 0.001**	**< 0.001**
DOM3	*13.33*	*(11– 16)*		11.21 ±4			*13.33*	*(12– 16)*		*14.67*	*(12– 17)*		**< 0.001**	**< 0.001**
DOM4	*15.50*	*(14– 17)*		13.5 ±3			*15.5*	*(15– 17)*		16.3 ±2			**< 0.001**	**< 0.001**
*OV QOL GH*	*7.00*	*(6– 8)*		*6*	*(4– 7)*		*8*	*(6– 8)*		*8*	*(7– 9)*		**< 0.001**	**< 0.001**
													** **	** **
PCL Total	*10.00*	*(9– 12)*		26.42 ±20			*9*	*(3– 19)*		*5*	*(2– 14)*		**< 0.005**	**< 0.001**
PTSD symptoms	16 (81%)			9(28%)			7 (4%)			0			**< 0.001**	**< 0.001**
CESD Total	*10*	*(5– 19)*		33.53 ±5			*9*	*(4– 13)*		*6*	*(3– 10)*		**< 0.001**	**< 0.001**
*STAI*													** **	** **
State Total	*38*	*(31– 47)*		53.16 ±10			*36*	*(30– 43)*		*32*	*(27– 36)*		**< 0.001**	**< 0.001**
Trait Total	*42*	*(35– 51)*		57.06 ±7			*40.5*	*(34– 47)*		*36*	*(33– 41)*		**< 0.001**	**< 0.001**
State anxiety symptoms	74 (37%)			28 (88%)			46 (28%)			0			**< 0.001**	**< 0.001**
Trait anxiety symptoms	81 (41%)			32 (100%)			49 (30%)			0			**< 0.001**	**< 0.001**

**Table 2.** Clinical and demographic characteristics of the study participants with state anxiety symptoms, compared to those without state anxiety symptoms and healthy controls.

	Total cohort (n = 198)			State anxiety (n = 74)			State anxiety ctrls (n = 124)			Healthy controls (n = 106)			State anxiety vs state anxiety ctrls	State anxiety vs healthy controls
	mean±SD			mean±SD			mean±SD			mean±SD				
	*median*	*(IQR)*		*median*	*(IQR)*		*median*	*(IQR)*		*median*	*(IQR)*			
	n	(%)		n	(%)		n	(%)		n	(%)		p-value	p-value
Age	*37*	*(30.0– 43.0)*		*37*	*(29.0– 42.8)*		*37.5*	*(30.0– 43.3)*		*38*	*(30– 44)*		NS	NS
Female	139	(70%)		53	(72%)		86	(69%)		71	(67%)		NS	NS
Weight	*65*	*(56.3– 73.0)*		*65*	*(57.3– 72.8)*		*65*	*(56.0– 73.0)*		*65*	*(57– 73)*		NS	NS
BMI	*22.9*	*(20.7– 24.9)*		*22.82*	*(20.9– 24.9)*		*22.9*	*(20.6– 24.8)*		*23.02*	*(21– 25)*		NS	NS
Autoimmune disease ever	24	(12%)		7	(9%)		17	(14%)		14	(13%)		NS	NS
IBD_IB_coeliacs ever	33	(17%)		10	(14%)		23	(19%)		21	(20%)		NS	NS
Periodontitis ever	44	(22%)		17	(23%)		27	(22%)		22	(21%)		NS	NS
Psych_meds_ever	58	(29%)		33	(45%)		25	(20%)		21	(20%)		**< 0.001**	**< 0.001**
Nicotine last 2 weeks	48	(24%)		27	(36%)		21	(17%)		19	(18%)		**< 0.005**	**< 0.01**
CTQ total	*31.0*	*(27.0– 39.0)*		*35*	*(30.0– 42.5)*		*30*	*(27.0– 36.0)*		*29*	*(27– 34)*		**< 0.001**	**< 0.001**
*WHOQOL*														
DOM1	*15.4*	*(13.7– 17.1)*		13.76 ±2.5			*16.57*	*(14.9– 17.7)*		*16.57*	(15– 18)		**< 0.001**	**< 0.001**
DOM2	*14.0*	*(12.0– 16.0)*		12.03 ±2.3			15.2 ±2.1			15.55 ±2			**< 0.001**	**< 0.001**
DOM3	*13.3*	*(10.7– 16.0)*		12.14 ±3.3			*14.67*	*(12.0– 17.3)*		*14.67*	*(12– 17)*		**< 0.001**	**< 0.001**
DOM4	*15.5*	*(14.0– 17.0)*		*14.25*	*(12.0– 16.0)*		*16*	*(15.0– 17.5)*		16.3 ±2			**< 0.001**	**< 0.001**
OV QOL GH	*7.0*	*(6.0– 8.0)*		*7*	*(5.3– 8.0)*		*8*	*(7.0– 9.0)*		*8*	*(7– 9)*		**< 0.001**	**< 0.001**
PCL Total	*10.0*	*(9.0– 12.0)*		*22*	*(7.0– 35.0)*		*5*	*(2.0– 14.3)*		*5*	*(2– 14)*		**< 0.001**	**< 0.001**
PTSD symptoms	16	(8%)		14 (19%)			2 (2%)			0			**< 0.001**	**< 0.001**
CESD Total	*10.0*	*(5.0– 19.0)*		*21.5*	*(13.0– 32.0)*		*8*	*(3.0– 12.0)*		*6*	*(3– 10)*		**< 0.001**	**< 0.001**
Depressive symptoms	32	(16%)		28	(38%)		4	(3%)		0			**< 0.001**	**< 0.001**
*STAI*													** **	** **
State Total	*38.0*	*(31.0– 46.8)*		*49*	*(45.0– 53.0)*		*32.5*	*(28.0– 37.0)*		*32*	*(27– 36)*		**< 0.001**	**< 0.001**
Trait Total	*42.0*	*(35.0– 51.0)*		52.04 ±8.2			*38*	*(33.0– 42.3)*		*36*	*(33– 41)*		**< 0.001**	**< 0.001**
Trait anxiety symptoms	81	(41%)		63	(85%)		18	(15%)		0			**< 0.001**	**< 0.001**

**Table 3.** Clinical and demographic characteristics of the study participants with trait anxiety symptoms, compared to those without trait anxiety symptoms and healthy controls.

		Total cohort (n = 198)			Trait anxiety (n = 81)			Trait anxiety ctrls (n = 117)			Healthy controls (n = 106)			Trait anxiety vs Trait anxiety ctrls	State anxiety vs healthy controls
		mean±SD			mean±SD			mean±SD			mean±SD				
		median	(IQR)		median(IQR)			median(IQR)			median(IQR)				
		n (%)			n (%)			n (%)			n (%)			p-value	p-value
Age	** **	*37.0*	*(30.0– 43.0)*		*37.0*	*(29.0– 41.0)*		*38.0*	*(30.0– 44.0)*		*38*	*(30– 44)*		NS	NS
Female	** **	139 (70%)			62	(77%)		77	(66%)		71 (67%)			NS	NS
Weight	** **	*65.0*	*(56.3– 73.0)*		*63.0*	*(56.0– 72.0)*		*65.0*	*(57.0– 74.0)*		*65*	*(57– 73)*		NS	NS
BMI	** **	*22.9*	*(20.7– 24.9)*		*22.8*	*(20.3– 24.9)*		*23.0*	*(32.3– 24.8)*		*23.02*	*(21– 25)*		NS	NS
	** **														
Autoimmune disease ever		24 (12%)			9 (11%)			15 (13%)			14 (13%)			NS	NS
IBD IB coeliacs ever		33.0 (17%)			8.0 (10%)			25.0 (21%)			21 (20%)			0.052	NS
Periodontitis ever		44 (22%)			20 (25%)			24 (21%)			22 (21%)			NS	NS
Time since COVID diag months		8.1 ±4.30			6.2 ±3.87			9.1 ±4.24			*2*	*(1– 3)*		**< 0.05**	**< 0.05**
Psych meds ever		58 (29%)			31 (38%)			27 (23%)			21 (20%)			**< 0.05**	**< 0.01**
	** **														
CTQ total	** **	*31.0*	*(27.0– 39.0)*		*35.0*	*(30.0– 41.0)*		*29.0*	*(27.0– 35.0)*		*29*	*(27– 34)*		**<0.001**	**< 0.001**
	** **														
*WHOQOL*															
DOM1		*15.4*	*(13.7– 17.1)*		13.9 ±2.46			*16.6*	*(14.9– 17.7)*		*16.57*	*(15– 18)*		**<0.001**	**< 0.001**
DOM2		*14.0*	*(12.0– 16.0)*		12.0 ±2.27			15.4±1.99			15.55 ±2			**<0.001**	**NS**
DOM3		*13.3*	*(10.7– 16.0)*		11.7 ±3.14			*14.7*	*(12.0– 17.3)*		*14.67*	*(12– 17)*		**<0.001**	**< 0.001**
DOM4		*15.5*	*(14.0– 17.0)*		14.1 ±2.35			*16.0*	*(15.0– 17.5)*		16.3 ±2			**<0.001**	**< 0.001**
OV QOL GH		*7.0*	*(6.0– 8.0)*		*7.0*	*(5.0– 7.0)*		*8.0*	*(7.0– 9.0)*		*8*	*(7– 9)*		**<0.001**	**< 0.001**
	** **													** **	** **
PCL Total	** **	*10.0*	*(9.0– 12.0)*		*22.0*	*(6.0– 35.8)*		*6.0*	*(2.0– 14.0)*		*5*	*(2– 14)*		**<0.001**	**< 0.001**
	** **													** **	** **
PTSD symptoms		16 (8%)			16	(20%)		0			0			**<0.001**	**< 0.001**
	** **													** **	
CESD Total		*10.0*	*(5.0– 19.0)*		*22.0*	*(13.0– 32.0)*		*6.0*	*(3.0– 10.0)*		*6*	*(3– 10)*		**<0.001**	**< 0.001**
	** **													** **	** **
Depressive symptoms		32 (16%)			32 (40%)			0 (0%)			0 (0%)			**<0.001**	**< 0.001**
	** **													** **	** **
*STAI*	** **													** **	** **
State Total		*38.0*	*(31.0– 46.8)*		48.4 ±9.03			32.8 ±6.76			*32*	*(27– 36)*		**<0.001**	**< 0.001**
Trait Total		*42.0*	*(35.0– 51.0)*		*53.0*	*(49.0– 58.0)*		*37.0*	*(33.0– 41.0)*		*36*	*(33– 41)*		**<0.001**	**< 0.001**
	** **													** **	
State anxiety symptoms		74 (37%)			63(78%)			11 (9%)			0 (0%)			**<0.001**	**< 0.001**

**Table 4.** Clinical and demographic characteristics of the study participants with PTSD symptoms, compared to those without PTSD symptoms and healthy controls.

	Total cohort (n = 198)			PTSD symptoms (n = 16)			PTSD ctrls (n = 182)			Healthy controls (n = 106)			PTSD vs PTSD ctrls	PTSD vs healthy ctrls
	mean±SD			mean±SD			mean±SD			mean±SD			p-value	p-value
	*median*	*(IQR)*		*median*	*(IQR)*		*median*	*(IQR)*		*median*	*(IQR)*			
	n (%)			n (%)			n (%)			n (%)				
Age** **	*37*	*(30.0– 43.0)*		35± 10.0			37	*(30.0– 43.0)*		*38*	*(30– 44)*		NS	NS
Female** **	139 (70%)			13 (81%)			126 (69%)			71 (67%)			NS	NS
Weight** **	*65*	*(56.3– 73.0)*		*59*	*(52.8– 64.5)*		*65*	*(57.0– 73.8)*		*65*	*(57– 73)*		NS	NS
BMI** **	*22.88*	*(20.7– 24.9)*		21.93 ±3.3			*22.96*	*(20.9– 25.0)*		*23.02*	*(21– 25)*		NS	NS
Autoimmune disease ever	24 (12%)			2 (13%)			22 (12%)			14 (13%)			NS	NS
IBD IB celiacs ever	33 (17%)			1 (6%)			33 (18%)			21 (20%)			NS	NS
Periodontitis ever	44 (22%)			5 (31%)			39 (21%)			22 (21%)			NS	NS
Time since COVID diag months	8.1 ±4.3			*5*	*(3.0– 5.0)*		8.43 ±4.2			*2*	*(1– 3)*		**< 0.05**	NS
Psych meds ever	58 (29%)			11 (69%)			47 (26%)			21 (20%)			**< 0.001**	**< 0.001**
CTQ total	*31*	*(27.0– 39.0)*		42.88 ±14.4			*31*	*(27.0– 38.8)*		*29*	*(27– 34)*		**< 0.05**	**< 0.005**
*WHOQOL*														
DOM1	*15.4*	*(13.7– 17.1)*		14.21 ±2.9			*15.43*	*(13.7– 17.1)*		*16.57*	*(15– 18)*		NS	**< 0.01**
DOM2	*14.0*	*(12.0– 16.0)*		11.46 ±1.9			*14.67*	*(12.7– 16.0)*		15.55 ±2			**< 0.001**	**< 0.001**
DOM3	*13.3*	*(10.7– 16.0)*		12.5 ±3.2			*13.33*	*(10.7– 16.0)*		*14.67*	*(12– 17)*		NS	**< 0.05**
DOM4	*15.5*	*(14.0– 17.0)*		14 ±2.6			*15.5*	*(14.5– 17.0)*		16.3 ±2			**< 0.05**	**< 0.05**
OV QOL GH	*7.0*	*(6.0– 8.0)*		6.25 ±1.6			*7*	*(6.0– 8.0)*		*8*	*(7– 9)*		**< 0.05**	**< 0.05**
PCL Total	*10.0*	*(9.0– 12.0)*		*43*	*(38.8– 46.3)*		*7*	*(2.0– 17.0)*		0			**< 0.001**	**< 0.001**
CESD Total	*10*	*(5.0– 19.0)*		25.13 ±9.6			*10*	*(5.0– 17.0)*		*6*	*(3– 10)*		**< 0.001**	**< 0.001**
Depressive symptoms	32 (16%)			9 (56%)			23 (13%)			0 (0%)			**< 0.001**	**< 0.001**
*STAI*** **													** **	** **
State Total	*38*	*(31.0– 46.8)*		52 (10%)			*37*	*(31.0– 45.0)*		*32*	*(27– 36)*		**< 0.001**	**< 0.001**
Trait Total	*42*	*(35.0– 51.0)*		55.25 ±7.2			*41*	*(35.0– 49.8)*		*36*	*(33– 41)*		**< 0.001**	**< 0.001**
State anxiety symptoms	74 (37%)			14 (88%)			60 (33%)			0 (0%)			**< 0.001**	**< 0.001**
Trait anxiety symptoms	81 (41%)			16 (100%)			65 (36%)			0 (0%)			**< 0.001**	**< 0.001**

Common variables that differed between all symptom groups and respective control groups included higher use of psychiatric medication, higher levels of childhood trauma, lower quality of life, and higher levels of other psychiatric symptoms. Those with state anxiety reported higher levels of smoking (Table 2) and those with trait anxiety and PTSD reported a more recent COVID-19 positive test compared to symptom controls (Tables 3 and 4). Participants with PTSD symptoms reported lower psychological quality of life compared to those without PTSD symptoms (Table 4).

### Lower diversity in individuals with symptoms of trait anxiety

**Figure 2.**
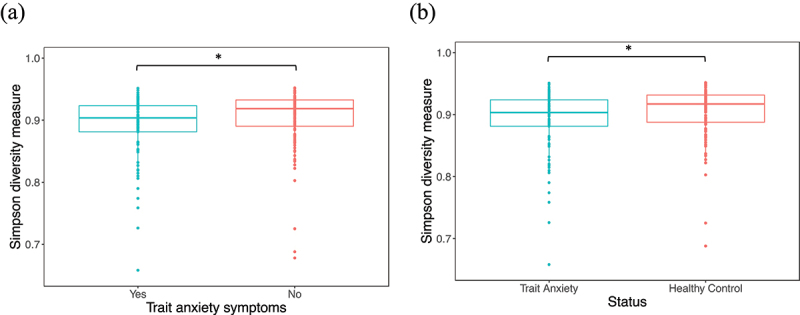
Simpson’s diversity index was significantly lower in individuals with (a) trait anxiety symptoms (*mdn* = 0.90) compared to those without (*mdn* = 0.92) (Wilcoxon rank-sum test, *p* = 0.016, *r =* 0.19, *n* = 198) and (b) compared to healthy controls (*mdn* = 0.92) (Wilcoxon rank-sum test, *p* = 0.02, *r =* 0.17, *n* = 198). The solid line indicates the median; the top and bottom of the boxes indicate the third and first quartiles, respectively. Whiskers indicate the 1.5 interquartile range (IQR) beyond the upper and lower quartiles and dots represent individual data points. Significance * for *p* ≤ 0.05.

Alpha diversity, as measured by Simpson’s diversity index, was lower in individuals with trait anxiety symptoms (median [*mdn*] = 0.9) compared to those without (*mdn* = 0.92) (Wilcoxon rank-sum tests, *p* = 0.016, *r =* 0.19, *n* = 198, Figure 2a) and compared to healthy controls (*mdn* = 0.92) (Wilcoxon rank-sum tests, *p* = 0.01, *r =* 0.17, *n* = 198; Figure 2b).

**Figure 3.**
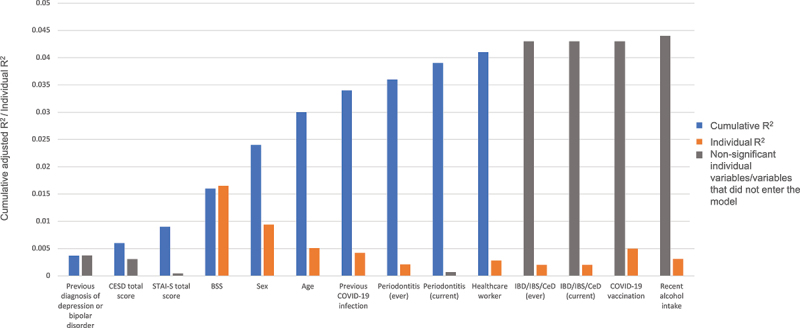
Cumulative effect sizes of variables on microbiome community variation (left blue bars; stepwise distance-based redundancy analysis (dbRDA) on genus-level Aitchison distance); individual effect sizes (assuming covariate Independence) (right Orange bars); variables with non-significant *p*-values for individual analyses (right gray bars) and the one variable (COVID-19 vaccination), that did not enter the dbRDA model (left gray bar).

There were no significant differences in genus- or phylum-level gut microbiome community composition (measured by Aitchison distance, an Euclidean distance on clr-transformed data), for any of the mental health symptoms of interest compared to their respective control groups. In addition, no differences in genus- or phylum-level gut microbiome community composition were noted based on the scores of the tests that evaluate these psychiatric symptoms. When evaluating the effect of other metadata variables on the ordination (using the *Capscale* (CAP) function and a permutational ANOVA), we found that age (CAP, *q* = 0.01, R^2^ = 0.005, *n* = 198), sex (CAP, *q* = 0.01, R^2^ = 0.009, *n* = 198), a previous COVID-19 infection (CAP, *q* = 0.02, R^2^ = 0.0043, *n* = 198), a COVID-19 vaccination (CAP, *q* = 0.01, R^2^ = 0.005, *n* = 198), the Bristol stool scale (BSS) (CAP, *q* = 0.01, R^2^ = 0.016, *n* = 198), ever being diagnosed with inflammatory bowel disease [IBD], irritable bowel syndrome [IBS], or Celiac disease [CeD] (IBD/IBS/CeD) (CAP, *q* = 0.07, R^2^ =0.002, *n* = 198), a current diagnosis of IBD/IBS/CeD (CAP, *q* = 0.08, R^2^ =0.002, *n* = 198), and alcohol intake in the last 2 weeks (CAP, *q* = 0.07, R^2^ =0.003, *n* = 198) influenced the genus-level fecal community composition (Figure 3, orange bars).

We used the *ordiR2step* function (a stepwise model selection using permutation tests) to test whether a group of mental health-related variables had an effect on the ordination, and found that CESD total score (ANOVA, *p* = 0.02, R^2^ = 0.006) + STAI-S total score (ANOVA, *p* = 0.04, R^2^ = 0.009) and a previous diagnosis of depression or bipolar disorder (ANOVA, *p* = 0.03, R^2^ = 0.004) had a significant effect on genus-level ordination (Figure 3, blue bars). The following group of metadata variables had an effect on the genus-level ordination: BSS (ANOVA, *p* = 0.002, R^2^ = 0.02) + sex (ANOVA, *p* = 0.002, R^2^ = 0.02) + age (ANOVA, *p* = 0.002, R^2^ = 0.03) + COVID positive test (ANOVA, *p* = 0.002, R^2^ = 0.03) + periodontitis (ever) (ANOVA, *p* = 0.04, R^2^ = 0.04) + periodontitis (current) (ANOVA, *p* = 0.03, R^2^ = 0.04) + healthcare worker (ANOVA, *p* = 0.03, R^2^ = 0.04) (Figure 3, blue bars).

### Associations between traumatic experiences, mental health outcomes, and relative taxonomic abundance

**Figure 4.**
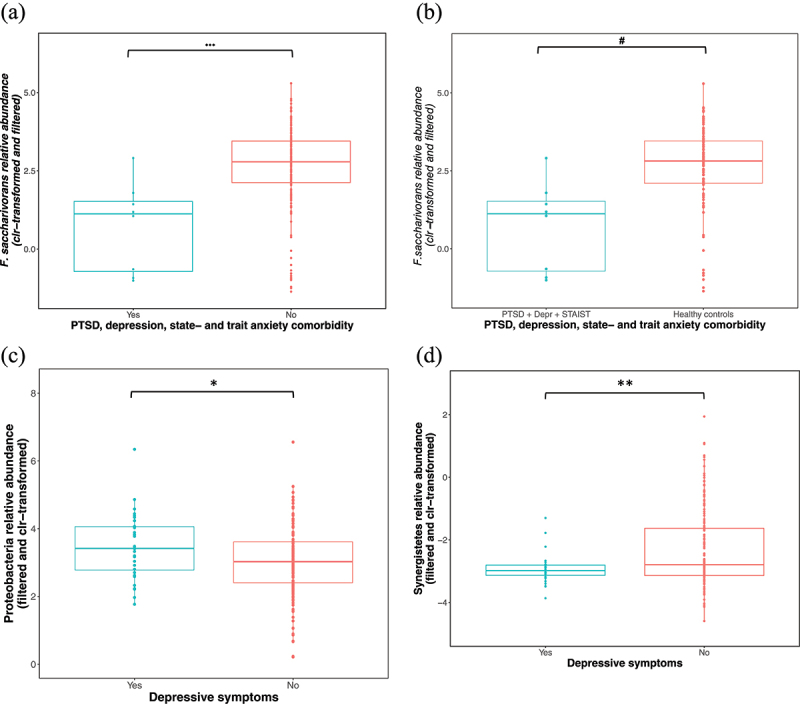
(a) *F. saccharivorans* was significantly lower in individuals with comorbid symptoms of PTSD + depression + state and trait anxiety (*mdn* = 1.12) (after correcting for main covariates) compared to individuals without these comorbid symptoms (*mdn* = 2.78) (GLM, *p* = 0.0001, *r =* 0.24, *n* = 198) and (b) compared to healthy controls (*mdn* = 2.82) (correction for confounding variables not possible) (Wilcoxon rank-sum tests, *q* = 0.1, *n* = 114). (c) Proteobacteria was higher in individuals with depressive symptoms (*mdn =* 3.42) (GLM, *p* = 0.02, *n* = 198) compared to those without (*mdn =* 3.02) whilst (d) Synergistetes were lower in those with depressive symptoms (*mdn* = −2.98) (GLM, *p* = 0.004, *n* = 198) compared to those without (*mdn* = −2.80). Sample sizes: PTSD + depression, state- and trait-anxiety symptoms Yes *n* = 8, PTSD + depression, state- and trait-anxiety symptoms No *n =* 190. Depressive symptoms Yes *n* = 32, Depressive symptoms No *n* = 166, Healthy controls *n* = 106. Solid lines indicate the median; the tops and bottoms of boxes indicate the third and first quartiles, respectively. Whiskers indicate the 1.5 IQR beyond the upper and lower quartiles. Dots represent individual data points. Abbreviations: clr – centered log-ratio, *r* = effect size. Significance * for *p* ≤ 0.05, ** for *p* ≤ 0.005, *** for *p* ≤ 0.0001, ^#^ for *q* ≤ 0.1. *Fusicatenibacter saccharivorans – F. saccharivorans*, posttraumatic stress disorder – PTSD, generalized linear model – GLM.

The relative abundance of *Fusicatenibacter saccharivorans* (*F. saccharivorans*) was significantly lower in individuals with comorbid PTSD + depression + state and trait anxiety symptoms (*n* = 8) (*mdn* = 1.12) compared to those without this comorbid state (*mdn* = 2.78) (*n* = 190: depressive symptoms *n* = 24, state anxiety symptoms *n* = 66, trait anxiety symptoms *n* = 73, PTSD symptoms *n* = 8, healthy controls *n* = 106) (Wilcoxon rank-sum test *q* = 0.09, *r =* 0.24, *n* = 198), and it remained significant after correcting for main covariates (age, sex, body mass index [BMI], inflammatory bowel disease [IBD], irritable bowel syndrome [IBS], Celiac disease [CeD], and BSS) using generalized linear models (GLMs) (GLM *p* = 0.0001) (Figure 4a). However, correction for additional covariates (current use of prescription medication, a previous COVID-19 infection, a COVID-19 vaccination, and alcohol intake in the last 2 weeks) rendered the model unreliable, potentially due to the small sample size of the comorbidity cohort. In addition, *F. saccharivorans* was also lower in this comorbid group (PTSD + depression + state and trait anxiety symptoms) compared to healthy controls (*n* = 106) (*mdn* = 2.82) (Wilcoxon rank-sum test *q* = 0.1, *r =* 0.3, *n* = 114) (Figure 4b), but also, in this case, correction for covariates rendered the model unreliable. Individuals with depressive symptoms had higher levels of the phyla Proteobacteria (*mdn =* 3.42) (GLM, *p* = 0.02, *r =* 0.16, *n* = 198) and lower levels of Synergistetes (*mdn* = −2.98) (GLM, *p* = 0.004, *r =* 0.17, *n* = 198) (Figure 4c,d), compared to those without depressive symptoms (Proteobacteria *mdn =* 3.02; Synergistetes *mdn* = −2.80), and it remained significant following correction for main and additional microbiome covariates. Since all individuals with depressive symptoms also experienced trait anxiety, trait anxiety total score was also corrected for in the GLM in addition to the additional covariates, and the associations remained significant (*p* = 0.03, *p* = 0.04 respectively), suggesting that the levels of Synergistetes could have been influenced by trait anxiety symptoms in those with depressive symptoms.

**Figure 5.**
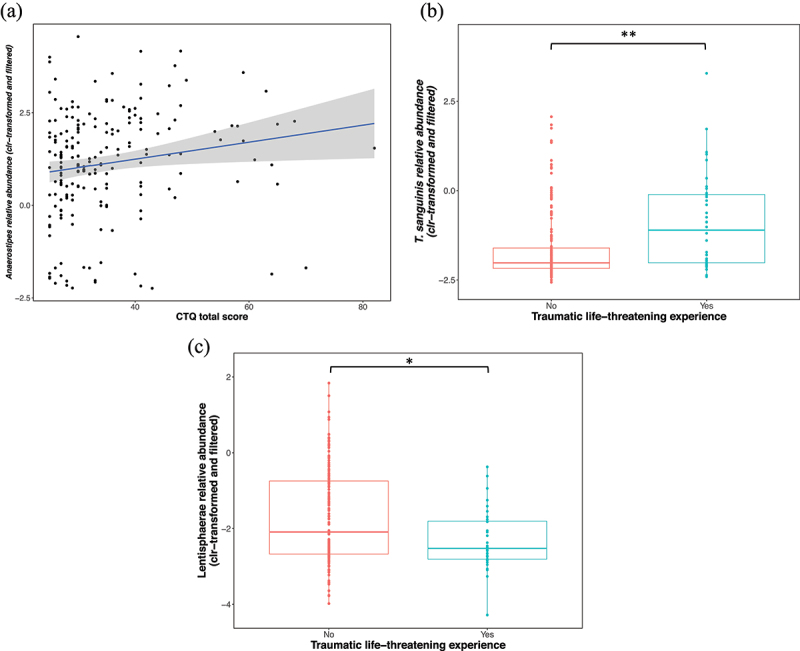
Positive relationship between the relative abundance of the *Anaerostipes* genus and CTQ total score (Spearman *r_s_* = 0.23; *p* ≤ 0.01, *n* = 198). (b) The relative abundance of *T. sanguinis* was higher in individuals who experienced life-threatening traumas (*mdn* = −0.65 versus *mdn* = −2.37) (GLM, *p* ≤ 0.001, *r =* 0.24, *n* = 198), and the relative abundance of Lentisphaerae was lower (GLM, *p* = 0.002, *r =* 0.20, *n =* 198) (*mdn* = −2.52 versus *mdn* = −2.09) compared to individuals unexposed to such traumas. Y-axes show the clr-transformed relative abundances of the taxa. For box plots, solid lines indicate the median; the top and bottom of boxes indicate the third and first quartiles, respectively. Whiskers indicate the 1.5 IQR beyond the upper and lower quartiles. Dots represent individual data points. Significance * for *p* ≤ 0.05, ** for *p* ≤ 0.005. Abbreviations: centered log-ratio – clr, Childhood Trauma Questionnaire – CTQ, *Turicibacter sanguinis – T. sanguinis*, effect size – *r.*

The relative abundance of the *Anaerostipes* genus was positively associated with the Childhood Trauma Questionnaire (CTQ) total score (Spearman *r_s_* = 0.23; *q* ≤ 0.1, *n* = 198) (Figure 5a); thus, higher levels were present in those who experienced more severe childhood trauma. This association remained significant after correcting for main and additional microbiome covariates (GLM, *p* ≤ 0.01, *n* = 198). Individuals who experienced life-threatening traumas (*n* = 36) had a significantly higher relative abundance of the *Turicibacter sanguinis* (*T. sanguinis*) species (*mdn* = −0.65) and significantly lower levels of the phylum Lentisphaerae (*mdn* = −2.52) compared to those who have not had such an experience (*n* = 162) (*T. sanguinis mdn* = −2.37; Lentisphaerae *mdn* = −2.09); these associations remained significant following correction for main and additional covariates (GLM, *p* = 0.0008, *r =* 0.24, and GLM, *p* = 0.002, *r =* 0.20 respectively, *n =* 198) (Figure 5b,c).

We also investigated whether there were differences in the relative taxonomic abundance between individuals with self-reported clinical diagnoses and those with symptoms of depression and anxiety. There were no statistically significant differences in taxonomic abundance between individuals with a self-reported current diagnosis of depression or bipolar disorder (*n* = 10) and individuals with high CESD scores (25 – 55) (*n* = 32). Furthermore, no differences were noted between individuals with a self-reported current diagnosis of an anxiety disorder (*n* = 27) and individuals with high STAI-T scores (≥ 60) (*n* = 14), or those with high STAI-S scores (≥ 55) (*n* = 16). For the PTSD symptom cohort, we could not compare those with a current diagnosis to those with symptoms, because data pertaining to previous diagnoses of PTSD were not available.

### Associations between COVID-19-related variables and relative taxonomic abundance

**Figure 6.**
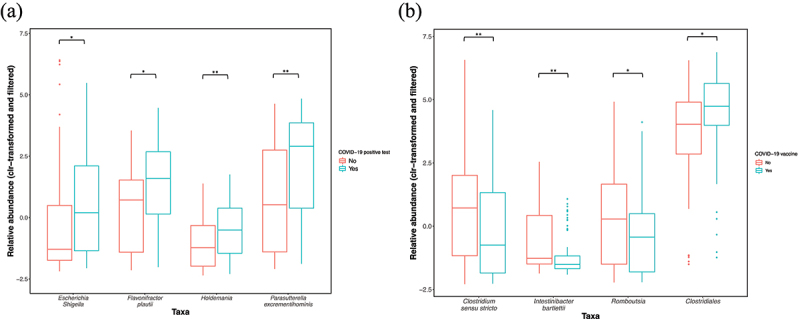
(a) Associations between a previous COVID-19 infection and the relative abundance of *Escherichia-Shigella* (*mdn* = 0.15 versus *mdn* = −1.36) (GLM, *p* = 0.004, *r* = 0.23, *n* = 198), *Parasutterella excrementihominis* (*mdn* = 2.85 versus *mdn* = 0.48) (GLM, *p* = 0.0003, *r* = 0.25, *n* = 198), *Flavonifractor plautii* (*mdn* = 1.52 versus *mdn* = 0.43) (GLM, *p* = 0.002, *r* = 0.21, *n* = 198) and *Holdemania* (*mdn* = −0.60 versus *mdn* = −1.29) (GLM, *p* = 0.0003, *r* = 0.24, *n* = 198). (b.) Associations between COVID-19 vaccine administration and the relative abundances of *Clostridium sensu stricto* (*mdn = −0*.79 versus *mdn =* 0.65) (GLM, *p* = 0.005, *r* = 0.22, *n* = 198), *Intestinibacter bartlettii* (*mdn = −1*.58 versus *mdn = −1*.33) (GLM, *p* ≤ 0.002, *r* = 0.3, *n* = 198), *Romboutsia* (*mdn = −0*.48 versus *mdn =* 0.22) (GLM, *p* = 0.01, *r* = 0.22, *n* = 198) and the Clostridiales order (*mdn =* 4.61 versus *mdn =* 3.77) (GLM, *p* = 0.01, *r* = 0.25, *n* = 198). Y-axes show the clr-transformed relative abundances of the taxa. The solid line indicates the median, lower and upper bounds of boxes indicate the first and third quartiles, respectively; whiskers indicate the 1.5 IQR beyond the upper and lower quartiles. Dots represent outlier data points. Sample sizes: previous COVID-19 infection YES *n* = 42, previous COVID-19 infection NO *n* = 156. COVID-19 vaccine administered YES *n* = 90, COVID-19 vaccine administered NO *n* = 108. Significance * for *p* ≤ 0.05, ** for *p* ≤ 0.005.

Following correction for the main microbiome covariates and additional covariates (COVID-19 vaccination, current use of prescription medication, and alcohol intake), individuals with a previous, confirmed COVID-19 infection (henceforth referred to as previous COVID-19 infection), had a significantly higher relative abundance of the genera *Escherichia-Shigella* (*mdn* = 0.15) (GLM, *p* = 0.004, *r* = 0.23, *n* = 198) and *Holdemania* (*mdn* = −0.60) (GLM, *p* = 0.0003, *r* = 0.24, *n* = 198) and the species *Parasutterella excrementihominis* (*P. excrementihominis*) (*mdn* = 2.85) (GLM, *p* = 0.0003, *r* = 0.25, *n* = 198) and *Flavonifractor plautii* (*F. plautii*) (*mdn* = 1.52) (GLM, *p* = 0.002, *r* = 0.21, *n* = 198) (Figure 6a), compared to those without a previous COVID-19 infection (*Escherichia-Shigella mdn* = −1.36, *Holdemania mdn* = −1.29, *P. excrementihominis mdn* = 0.48, *F. plautii mdn* = 0.43). Following correction for main and additional microbiome covariates (previous COVID-19 infection, current use of prescription medication, and alcohol intake), individuals who received a COVID-19 vaccination had a higher relative abundance of the Clostridiales order (*mdn =* 4.61 versus *mdn =* 3.77) (GLM, *p* = 0.01, *r* = 0.25, *n* = 198), and lower levels of the genera *Romboutsia* (*mdn = −0*.48 versus *mdn =* 0.22) (GLM, *p* = 0.01, *r* = 0.22, *n* = 198), *Clostridium sensu stricto* (*mdn = −0*.79 versus *mdn =* 0.65) (GLM, *p* = 0.005, *r* = 0.22, *n* = 198), and the *Intestinibacter bartlettii* species (*mdn = −1*.58 versus *mdn = −1*.33) (GLM, *p* = 0.002, *r* = 0.3, *n* = 198) (Figure 6b) compared to unvaccinated individuals.

### Associations between health, well-being, and lifestyle-related variables and relative taxonomic abundance

**Figure 7.**
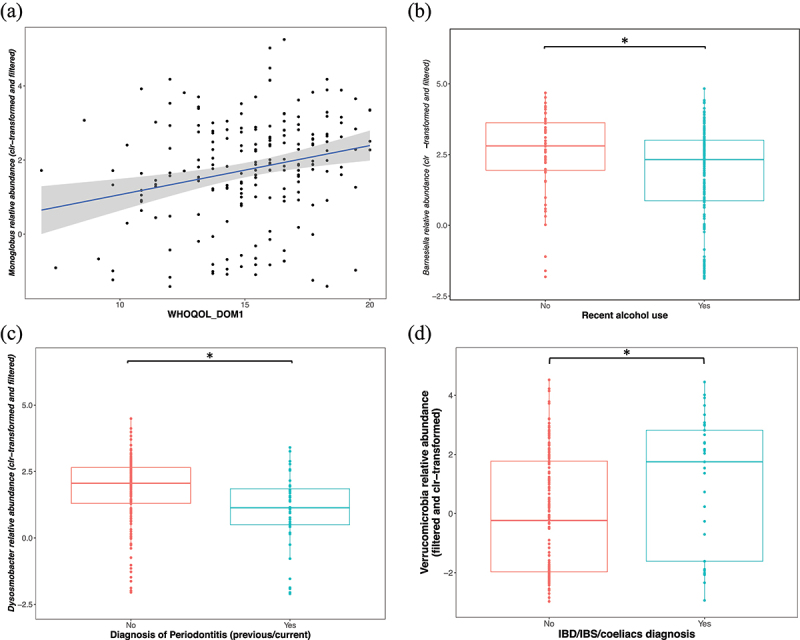
(a) Positive correlation between *Monoglobus* abundance and WHOQOL domain 1 scores (Spearman *r_s_* = 0.26; GLM *p* = 0.01, *n* = 198). (b) Negative associations between recent alcohol use and the relative abundance of *Barnesiella* (*mdn* = 2.32 versus *mdn* = 2.8) (GLM, *p* = 0.03, *r =* 0.2, *n* = 198), and (c) between periodontitis diagnosis (current and/or previous) and the relative abundances of *Dysosmobacter* (*mdn* = 1.13 versus *mdn* = 2.06) (GLM, *p* = 0.002, *r =* 0.3, *n* = 198). (d) Lower relative abundance of Verrucomicrobia in individuals with a current/prior diagnosis of IBD/IBS/CeD (*mdn* = 1.75 versus *mdn* = 0.23) (GLM, *p* = 0.03, *r =* 0.2, *n* = 198). Y-axes show the clr-transformed relative abundances of the taxa. The solid line indicates the median, lower and upper bounds of boxes indicate the first and third quartiles, respectively; whiskers indicate the 1.5 interquartile range IQR beyond the upper and lower quartiles. Dots represent individual data points. Sample sizes: Alcohol intake YES *n* = 146, Alcohol intake NO *n* = 52, Periodontitis diagnosis YES *n* = 44, Periodontitis diagnosis NO *n* = 154. IBD/IBS/CeD YES *n =* 33, IBD/IBS/CeD NO *n* = 165. Significance * for *p* ≤ 0.05. Celiac disease – CeD, inflammatory bowel disease – IBD, irritable bowel syndrome – IBS, World Health Organization Quality Of Life scores for domain 1 (physical health) – WHOQOL_DOM1.

The relative abundance of the *Monoglobus* genus was positively associated with the World Health Organization Quality Of Life questionnaire (WHOQOL) domain 1 scores (physical health), and it remained significant following correction for additional covariates (Spearman *r_s_* = 0.26; GLM *p* = 0.01, *n* = 198) (Figure 7a). The relative abundance of the genus *Gemmiger* was lower in individuals who reported current prescription medication use (*mdn* = 4.54 versus *mdn* = 5.04), and it remained significant following correction for main microbiome covariates (GLM, *p* = 0.02, *r* = 0.24, *n* = 198), but when correcting for additional covariates, no significant difference was noted. Individuals reporting alcohol consumption (in the last two weeks prior to the study) had a lower relative abundance of *Barnesiella* (*mdn* = 2.32) compared to those who did not report alcohol use (*mdn* = 2.8) (GLM, *p* = 0.03, *r =* 0.2, *n* = 198), and it remained significant following correction for additional microbiome covariates (a previous COVID-19 infection, COVID-19 vaccine, and current use of prescription medication) (Figure 7b).

Individuals with a diagnosis of periodontitis (previous or current, based on self-report medical questionnaire) had a significantly lower relative abundance of *Dysosmobacter* (*mdn* = 1.13) compared to those without a diagnosis (*mdn* = 2.06), and it remained significant following correction for additional covariates (GLM, *p* = 0.002, *r =* 0.3, *n* = 198) (Figure 7c). Finally, individuals with a current/prior diagnosis of IBD/IBS/CeD (based on self-report medical questionnaire) had a higher relative abundance of the Verrucomicrobia phyla (*mdn* = 1.75) compared to those without a diagnosis (*mdn* = −0.23), and it remained significant following correction for main and additional covariates (GLM, *p* = 0.03, *r =* 0.2, *n* = 198) (Figure 7d).

## Discussion

This study identified associations between the gut microbiome and mental health symptoms, traumatic experiences, well-being, and health-related symptoms in a naturalistic Spanish cohort in the aftermath of the COVID-19 pandemic. A recent study reported a steep global rise in the prevalence of anxiety and depression following the COVID-19 pandemic,^26^ further emphasizing the importance to prioritize mental health research and investigations into factors that play a role in these disorders. We, therefore, anticipate that there could be many undiagnosed cases in the general public and that measuring self-report symptoms could provide valuable insights into the mental health status of the population. Although our participants did not undergo a clinical assessment to formally diagnose anxiety, depression, or PTSD, validated questionnaires were used to assess these symptoms. Mental health disorders are characterized by the heterogeneity and complexity of symptoms; patients diagnosed with the same psychiatric disorder may present with different sets of symptoms in their clinical presentation,^27^ and it is important to study these disorders in this context. It is worth noting that symptoms inform diagnoses and treatment strategies,^28^ and oftentimes associations with biological markers correlate more strongly with symptoms and symptom dimensions as opposed to rigid diagnostic criteria.^29^

Simpson’s diversity index characterizes the number (species richness) and distribution (evenness) of taxa in a community. Our results showed that individuals with trait anxiety symptoms had lower diversity, however, the effect size was relatively small. Although several studies did not detect differences in alpha diversity measures in patients with self-reported generalized anxiety disorder (GAD) ^30,31^ or anxiety symptoms,^32,33^ our results correlate with findings of lower alpha diversity in patients with GAD compared to healthy controls ^31^ and in participants with IBS and high anxiety/depressive symptoms compared to controls and IBS-only cohorts.^34^ Lower alpha diversity has been reported in several disease cohorts relative to controls, including certain mental health disorders.^35,36^ Higher diversity is generally believed to signify microbial functionality and stability and was regarded to be more favorable for the host,^37^ which suggests that the participants with trait anxiety symptoms had a less favorable microbial profile compared to those without, and healthy controls. However, researchers have warned that this assumption oversimplifies complex mechanisms involved in community diversity and that the diversity measures should rather serve as a starting point for further investigations of ecological mechanisms.^38^

In our cohort, none of the mental health variables independently influenced the overall genus-level microbial composition (beta-diversity), which correlates with previous findings in MDD,^39^ PTSD,^20,40^ and anxiety.^30^ Our analysis of the possible effect of several mental health variables on microbial composition (assuming covariate dependence) revealed that CESD total score + STAI-S total score + a previous diagnosis of depression or bipolar disorder had a significant effect on genus-level ordination. Independent variables that influenced the overall genus-level microbial composition included age, sex, BSS, a previous COVID-19 infection, and a COVID-19 vaccination, ever being diagnosed with periodontitis, being a healthcare worker, having a diagnosis of IBD/IBS/CeD (ever/current), and recent alcohol intake. The following group of variables influenced the genus-level ordination (assuming covariate dependence), namely BSS + sex + age + previous COVID-19 infection + periodontitis diagnosis (current/ever) + being a healthcare worker. Other researchers also noted that some of these variables affected overall microbial diversity, including age,^41^ sex,^42^ BSS,^43^ IBD/IBS/CeD diagnosis,^44^ alcohol consumption,^45^ and COVID-19 infection.^46^ Limited human gut microbiome data in periodontitis patients are currently available, however, a rodent study showed that *P. gingivalis* infection was associated with differences in community structure. As hypothesized by these authors, *P. gingivalis* infection, the main cause of periodontitis, may have encouraged the growth of a particular set of taxa in the gut.

Although there were no associations between mental health symptoms and the global microbiome composition (in terms of beta diversity metrics), we detected associations between mental health symptoms and the relative abundance of particular taxa. Individuals with comorbid symptoms of state and trait anxiety, depression and PTSD had decreased levels of *F. saccharivorans*, which correlated with studies reporting a negative correlation between the abundance of *F. saccharivorans* and depressive symptoms.^47,48^ *Fusicatenibacter* produces lactate, formate, acetate, and succinate as fermentation end products from glucose ^47^ as well as the anti-inflammatory short-chain fatty acid (SCFA), butyrate.^49^ Butyrate is an important regulator of transepithelial fluid transport; it reduces mucosal inflammation and oxidative stress; strengthens the epithelial defense barrier, and moderates intestinal motility and visceral sensitivity (as reviewed by Canani et al.) .^50^ Furthermore, the abundance of *Fusicatenibacter* was found to be negatively correlated with serum levels of pro-inflammatory cytokines (including IL-6, TNF-α, and IL-1β) ^51^ and positively associated with serum levels of acetylcarnitine – an acetylated form of L-carnitine, synthesized *in vivo* and supplemented by diet, which has antidepressant properties and regulates sleep rhythm and quality.^52^

We, therefore, hypothesize that decreased levels of *F. saccharivorans* in individuals with comorbid anxiety, depressive and PTSD symptoms, may result in reduced levels of butyrate, which could compromise the epithelial gut lining and result in mucosal inflammation and increased circulating pro-inflammatory cytokines (due to bacteria and toxins that enter systemic circulation via a compromised gut epithelial lining). Furthermore, in individuals with these comorbid symptoms, it is possible that reduced *F. saccharivorans* correlated with lower levels of the antidepressant acetylcarnitine, which may have facilitated and/or exacerbated anxiety symptoms (since most antidepressants also have anti-anxiety effects) .^53^ These hypotheses however need to be tested in future studies and additional research is needed to determine whether the depletion of *F. saccharivorans* plays a causative role in the presentation of these symptoms. *F. saccharivorans* can easily be modulated by the addition of resistant maltodextrin ^54^ to the diet, and could therefore provide a safe, easy, and cost-effective means of improving anxiety as well as comorbid symptoms.

A higher relative abundance of Proteobacteria and a lower relative abundance of Synergistetes phyla were observed in individuals with depressive symptoms. A previous study also reported higher levels of Proteobacteria in active and responded MDD patients ^55^ and preclinical findings showed that exposure to chronic unpredictable mild stress,^56^ chronic subordinate colony housing ^57^ and immobilization stress ^58,59^ resulted, not only in depressive behaviors but also higher levels of Proteobacteria. This suggests that the higher abundance of Proteobacteria we observed, could be a direct consequence of stress exposure. Furthermore, a higher abundance of Proteobacteria is generally associated with pro-inflammatory states,^60^ which is often observed in individuals with mental health conditions.^61^ It is therefore plausible that previous exposures to stressful conditions promoted the expansion of Proteobacteria with subsequent pro-inflammatory consequences, and that this may have contributed to the later presentation of depressive symptoms in this cohort. Campo and colleagues discovered that a probiotic preparation of *Lactobacillus reuteri* improved the digestive health of cystic fibrosis patients by reducing proteobacterial populations in the gut,^62^ therefore, future studies could investigate how reducing the levels of Proteobacteria might influence depressive symptoms.

Data on Synergistetes in mental health conditions are relatively scarce. Lower levels of Synergistetes have been noted in older adults with insomnia,^63^ which is highly comorbid with depression.^64^ In addition, lower levels of Synergistetes have been detected in patients with IBS + high anxiety versus IBS + low anxiety.^14^ A better taxonomic resolution on genus- or species-level might enable stronger conclusions.

Trauma exposure, especially during developmental stages, is a strong risk factor for the development of mental health disorders.^65^ Living conditions and environmental exposures during childhood have also been shown to have long-lasting effects on the adult gut microbiome.^17^ Higher levels of childhood trauma were reported by all symptom groups in our cohort. Higher levels of childhood trauma were associated with an increased relative abundance of *Anaerostipes*. This finding correlates with data from rodent models; one model of early-life stress (ELS) ^66^ also detected higher levels of *Anaerostipes* following ELS exposure ^67^ and another detected higher levels of *Anaerostipes* in response to chronic restrained stress,^68^ suggesting a causal effect of trauma on the abundance of *Anaerostipes*. Furthermore, a higher abundance of *Anaerostipes* has been observed in MDD patients ^69^ and individuals with low mood.^70^ *Anaerostipes* is a butyrate-producing taxon;^71^ increased butyrate production is generally associated with improved intestinal epithelial function and immune profiles. However, *Anaerostipes* belongs to the Firmicutes phylum, and increased levels have previously been linked to inflammatory processes.^72^ It is possible that particular *Anaerostipes* species and strains could have different roles in and associations with stress exposure, immune reactivity, and intestinal integrity. Future studies should elucidate the mechanisms whereby stress exposure facilitates the expansion of *Anaerostipes* and the subsequent functional consequences.

Individuals who experienced a life-threatening traumatic event had a higher relative abundance of *T. sanguinis*, which strongly corresponds with results from a preclinical study that used an aggressor-exposed social stress mouse model that mimics warzone conflicts, where random life-threatening interactions occur between aggressive resident mice and naïve intruder mice. This study also reported higher levels of *Turicibacter* in naïve intruder mice shortly after threatening aggressor exposure.^73^ *T. sanguinis* is a common gut microbe that has the ability to signal to nearby intestinal cells to release serotonin, which subsequently promotes the expression of growth- and survival-related genes, enabling the microbe to colonize the host’s gut.^74^ Treating mice with a serotonin reuptake inhibitor (SSRI) (one of the main treatments for trauma-related disorders, such as PTSD) blocks the serotonergic uptake, and impedes the colonization of *T. sanguinis*. Up to 90% of the body’s serotonin originates from gut cells, and 50% of this production is regulated by a metabolite from *T. sanguinis*.^74^ It is, therefore, possible that higher levels of *T. sanguinis* result in altered serotonin levels in the gut, which may contribute to the gastrointestinal (GI) symptoms often experienced by patients using SSRIs. Under normal conditions, peripheral serotonin cannot cross the blood-brain barrier (BBB), however, altered levels in the gut could influence the tryptophan metabolism via serotonin synthesis and kynurenine degradation pathways,^75^ which could ultimately influence central nervous system functions. The data suggest that threatening and stressful exposures result in altered levels of serotonin via HPA axis activation (in the case of PTSD, HPA axis dysregulation), which in turn, influences the abundance of *T. sanguinis*. However, future studies should investigate this in more detail, to unravel the true cause and consequence.

We also detected lower levels of the Lentisphaerae phyla in individuals who experienced a life-threatening traumatic event, which is in line with an earlier study that reported a lower relative abundance of a consortium of three phyla in PTSD patients compared to trauma-exposed controls, where Lentisphaerae was part of this consortium. A lower abundance of Lentisphaerae has also been associated with global sleep dysfunction,^76^ which is highly prevalent in individuals exposed to stress.^77^

All of our symptom cohorts had significantly lower quality of life scores compared to control groups, as would be expected. We observed a positive correlation between *Monoglobus* and physical quality of life. The only species characterized to date is *Monoglobus pectinilyticus* (*M. pectinilyticus*), which possesses a specialized glycobiome for degrading pectin, a major polysaccharide that forms part of the plant cell wall.^78^ A study that employed a six-day, lifestyle-based immersion intervention program (consisting of daily nutrition education; 100% plant-based, whole food meals with minimal sugar, salt, and oil; cardiopulmonary exercise; and stress management classes) in individuals with high atherosclerotic cardiovascular disease risk, found that individuals with the greatest decreases in BMI, exhibited an increase in *Monoglobus* levels, and this genus was also positively correlated with changes in diastolic blood pressure and glucose and negatively associated with changes in total:HDL ratio.^79^ It is possible that the positive correlation we observed between physical quality of life and *Monoglobus* was driven by plant-rich diets, which promoted the growth of pectin-degrading species such as *M. pectinilyticus*, with subsequent beneficial effects, especially cardiometabolic health. However, the exact mechanisms and pathways of this relationship need to be investigated, and could once again offer interesting avenues to explore in order to promote general health and well-being.

Recent research suggests a role for the oral-gut-brain axis in mental health conditions. Comorbidity of periodontitis and mental health conditions have been observed, where mental health conditions, as well as periodontitis, were characterized by a pro-inflammatory state. Periodontitis may therefore be a risk factor for the later development of anxiety, mood, and stress-related disorders,^80^ and *vice versa* (as reviewed by Martínez et al. ^81^). Furthermore, a recent preclinical study confirmed that *P. gingivalis* affects brain areas related to anxiety, by inducing neuroinflammation.^81^ Although we did not see a higher prevalence of periodontitis diagnoses in the individuals with mental health symptoms, it is possible that undiagnosed cases were present in our cohort. Our results did show that a previous or current diagnosis of periodontitis influenced the overall genus-level composition, and that diagnosis of periodontitis (previous and/or current) was associated with a lower relative abundance of *Dysosmobacter* – a novel butyrate-producing bacterium from the Ruminococcaceae family. The species *Dysosmobacter welbionis* (*D. welbionis*) is present in about 70% of the general population and its abundance was inversely correlated with BMI, glycemia, and glycated hemoglobin in overweight and obese participants with a metabolic syndrome.^82^ A mouse model showed that daily oral gavage of live *D. welbionis* J115T resulted in a partially protective effect against fat mass gain and diet-induced obesity, with improved glucose tolerance and lower insulin resistance.^82^

Although no literature is available regarding the role of *Dysosmobacter* in periodontitis, periodontitis has been associated with and implicated in the etiology and pathophysiology of diseases like diabetes mellitus and cardiovascular disease.^83,84^ Higher levels of *D. welbionis*, possibly originating from the oral cavity, may have protected obese individuals with metabolic syndrome against certain metabolic derangements, by improving glucose tolerance, lowering insulin resistance, and reducing white adipose tissue hypertrophy and inflammation.^82^ In our cohort, lower levels of *Dysosmobacter* were present in the gut microbiome of individuals with periodontitis, and this may have correlated with increased levels of inflammation, which is also typical in periodontitis. Future studies should investigate the levels of *Dysosmobacter* in the gut and oral cavity of individuals with periodontitis. In addition, studies that investigate the oral-gut-brain axis in anxiety and depression are warranted.

We also detected a lower relative abundance of Verrucomicrobia in individuals with a current/prior diagnosis of IBD/IBS/CeD, which is consistent with previous findings in IBS.^85^ Anxiety and depressive symptoms are common in patients with IBD/IBS/CeD.^86^ When these mental health symptoms are not addressed, intestinal symptoms are further exacerbated. Patients with mental health symptoms also commonly present with GI symptoms, however, few clinical studies have disentangled the complex relationship between these comorbidities. One study reported that 70% of adults with IBD and a lifetime history of an anxiety or mood disorder had a first episode of an anxiety disorder that preceded the IBD diagnosis by 10 years or more and 8% developed anxiety two or more years after the onset of IBD, suggesting anxiety symptoms likely predate IBD. In terms of depression, 54% of individuals with IBD and a lifetime history of an anxiety or mood disorder had an onset of depression two or more years before the onset of IBD, while 23% developed depression two or more years following IBD onset, suggesting a risk of depressive symptoms before and after GI disease onset.^86^ Improving GI symptoms might therefore also improve symptoms of anxiety and depression and more studies are needed to determine how the microbiome can be targeted to improve comorbid symptoms of anxiety and/or depression and IBD/IBS/CeD.

COVID-19 had a significant effect on the gut microbiomes of participants; COVID-19 infection and vaccination influenced beta diversity and were also associated with the abundance of particular taxa. Interestingly, however, infection and vaccination were associated with distinct sets of taxa. A previous COVID-19 infection was associated with higher relative abundances of *Escherichia-Shigella, P. excrementihominis, F. plautii*, and *Holdemania*. Another study also reported elevated levels of *Escherichia-Shigella* in COVID-19 patients, which was associated with increased pro-inflammatory cytokines ^87^ and Zhou and colleagues detected elevated levels of the inflammation-related *F. plautii* in recovered COVID-19 patients compared to uninfected controls.^88^ Individuals who received a COVID-19 vaccine had higher levels of the Clostridiales order, and lower levels of *Romboutsia, Clostridium sensu stricto, Acidaminococcus*, and *I. bartlettii*. Another study did report lower levels of *Romboutsia* in COVID-19 patients compared to healthy controls,^89^ however, additional correlations with previous research are hampered by the lack of data on gut microbiome alterations associated with COVID-19 vaccinations. In our cohort, participants received different types of vaccines, some also received boosters of a different kind, which impeded stratification according to vaccination type. Infection with SARS-CoV2 has been associated with changes in the gut microbiome, especially the abundance of taxa associated with inflammatory processes. It is plausible that the pro-inflammatory state induced by a SARS-CoV2 infection could be further compounded by an altered gut microbiome, and this together with the stress during the time of the pandemic, may have created a perfect storm for the promotion of symptoms such as anxiety and depression.

Amongst the other metadata variables that were associated with gut microbiome composition, we found that alcohol consumption affected the genus-level ordination and was also associated with lower relative abundances of *Barnesiella*. Findings from Leclercq also revealed negative correlations between ethanol levels and the relative abundances of *Barnesiella*.^90^

Our findings should be interpreted in the context of particular limitations. Our study had a cross-sectional, naturalistic design, and participants were recruited from the general population. All data (except the microbiome data) is self-reported and is therefore susceptible to self-report bias, inaccuracies in recall, or misunderstanding of questions. Numerous factors can influence the composition of the gut microbiome (including dietary, lifestyle, genetic, environmental, and other health-related variables), all of which cannot be corrected as covariates in the analyses. Although GLMs enable us to correct for microbiome covariates, an excess of covariates results in a loss of statistical power and may cause overfitting of the data.^91^ We did however correct for main microbiome covariates identified by large-scale population-based studies ^23^ (age, sex, BMI, previous diagnosis of IBD/IBS/CeD, BSS) as well as additional microbiome covariates which had an effect in our cohort, namely current use of prescription medication, a previous COVID-19 infection, a COVID-19 vaccination, and alcohol intake in the last 2 weeks. Furthermore, study participants are only assessed at a single time point and therefore conclusions regarding longitudinal microbial composition and its impact on and associations with symptoms of anxiety and depression cannot be inferred.

Although the gut microbiota is amenable to change, especially during different life stages, after infections or antibiotic use, and in response to significant dietary interventions,^92^ the human gut microbiota is relatively stable over time:^92–95^ up to 60% of strains are reported to remain stable for up to five years, and several are possibly stable for decades.^96^ We, therefore, anticipate that within this short space of time (maximum four days between stool sample collection and questionnaire completion), the bacterial taxa we report on were indeed correlated to the mental health outcomes of the participants at that particular time. Additional investigations are required to determine how longitudinal changes in the gut microbiome could influence symptom presentation and *vice versa*.

## Conclusion

This investigation into the fecal microbiome of a Spanish cohort identified taxa that are associated with symptoms of depression as well as comorbid states of PTSD, depression, and anxiety. In addition, we identified taxa that were associated with trauma exposure, a known risk factor for the later development of mental health conditions. The relative abundance of certain gut microbial taxa was associated with well-being and health-related variables that could impact mental health, such as physical quality of life and diagnoses of IBD/IBS/CeD. Although the causality and directionality of these interactions cannot be inferred, our analyses took into consideration the compositionality of microbiome data and potential confounding effects. Since the abundance of *F. saccharivorans* (associated with comorbid symptoms of PTSD, depression, and anxiety), Proteobacteria (associated with depressive symptoms), and *Monoglobus* (associated with physical quality of life) can easily be modulated, these findings can contribute to future intervention studies to improve anxiety and depressive symptoms and promote general health and well-being.

## Materials and Methods

### Study participant recruitment, evaluation, and enrollment

The PsicoBioma research study has been carried out in accordance with The Code of Ethics of the World Medical Association (Declaration of Helsinki) for experiments involving humans and the data obtained was processed in accordance with the Spanish Organic Law 3/2018, on the Protection of Personal Data and the guarantee of digital rights (BOE 16673 of 6 Dec 2018) and its 17th Additional Provision. The study was approved by the Ethics Committee of Hospital Clínico San Carlos (Madrid) (C.P. PSQ-19-2 – C.I. 19/474-E). The study was conducted at a time when certain COVID-19 restrictions were still in place in Spain and hospital staff was inundated. A naturalistic, online study design was therefore implemented that utilized validated, self-report questionnaires. Purposive recruitment was used in the general population in Spain, using social media, web, and print advertisements. Recruitment criteria focused on: (1) Individuals who have previously been diagnosed with depression and/or an anxiety disorder and/or PTSD; (2) individuals who experienced symptoms of depression and/or anxiety and/or PTSD (these might include possible undiagnosed individuals with significant symptoms, especially in light of the increased prevalence of mental health disorders following the pandemic) as well as (3) healthy controls (described as individuals who, at the current time, did not experience the aforementioned mental health symptoms and have a sense of mental and general well-being).

Online, written informed consent was obtained from all research participants. Inclusion criteria: individuals had to reside in Spain, be at least 18 years of age, be able to read and understand Spanish, and meet the aforementioned recruitment criteria. Exclusion criteria: a prior or current diagnosis of any *other* major psychiatric disorder, other than anxiety, depression, and PTSD (including psychotic disorders, personality disorders, and neurodegenerative disorders); diarrhea within the past week (before stool sampling) or antibiotic use within the previous 6 months.

### Demographic and clinical data

Demographic and clinical data were collected using a secure online questionnaire that included structured demographic and medical history questionnaires designed for the PsicoBioma study (March 2021 – Jan 2022) (all data are therefore self-reported). Psychological assessments were all based on standardized self-report questionnaires validated for the Spanish population. As the questionnaires were not clinician-administered, the present study reports on symptoms rather than diagnoses. Depressive symptoms were evaluated using the CESD scale and state and trait anxiety symptoms using the STAI. Trauma exposure was evaluated with the PCL-5 as well as the CTQ-Short Form. Finally, quality of life was measured using the WHOQOL. The following criteria were used to determine the presence of psychiatric symptoms:

PTSD symptoms: PCL-5 score > 33 and the presence of more than 3 symptom clusters. ;^97^ state anxiety symptoms: STAI-S scores > 41; trait anxiety symptoms: STAI-T scores > 45; depressive symptoms: CESD scores ≤ 15 indicated no/low, 16 – 24 mild and 25 – 55 significant depressive symptoms.^98^ The total score and specific sub-scores of the CTQ Short Form ^99^ were used to evaluate the severity of childhood maltreatment. Individuals who did not meet the cutoff criteria described above for a particular mental health outcome were classified as a control for that particular symptom (therefore depressive controls, state- and trait anxiety controls, and PTSD controls, however, they may have been classified as having one/more of the other mental health symptoms). Individuals who did not meet the cutoff criteria for *all* the mental health outcomes were classified as healthy controls.

### Bacterial DNA extraction and generation of 16S rRNA gene V3-4 amplicons

Stool samples were collected by participants within four days of completing the online questionnaire, to ensure clinical and microbial data are comparable. Microbial DNA was extracted from stool samples homogenized in stool DNA-stabilizing buffer, using the PSP Spin Stool DNA Plus Kit (STRATEC Molecular, Birkenfeld, Germany) according to the manufacturer’s instructions (Protocol 2). Amplicons derived from the bacterial 16S rRNA gene V3-4 amplicons were generated using the 341 forward (5’- *CCTACGGGNGGCWGCAG*-3’) and 805 reverse (5’-*GACTACHVGGGTATCTAATCC*-3’) primer pair, as previously described.^100^

### 16S rRNA gene sequence and data preparation

Pooled 16S rRNA V3-4 gene amplicons were normalized and sequenced by *Laragen, Inc*. (California, USA) using the Illumina MiSeq® platform. Briefly, the 16S rRNA gene library concentration was measured using the Qubit 4 Fluorometer (Theromofisher, USA). The 16S rRNA gene library was sequenced with 300-bp paired-end reads on an Illumina MiSeq® sequencing system using a Nextera XT Index Kit v2 (600 cycles; Cat. No. TG-31-1096, Illumina Inc., San Diego, CA, USA), generating about 85 000 reads per sample. FASTQ files for forward and reverse reads and the index (barcode) read were generated.

Quality control of the FASTQ sequencing files was performed using *fastqc* (source code: https://github.com/s-andrews/FastQC) and *multiqc* (source code: https://github.com/ewels/MultiQC). Raw sequence reads were filtered using the Divisive Amplicon Denoising Algorithm 2 analysis package in R ^101^ (*dada2* version 1.12.1) with default parameters:^102^ expected error threshold of 2, trimming 17 nucleotides from the start of the forward reads to remove the 341F primer, and trimming 21 nucleotides from the start of the reverse reads to remove the 785R primer. Filtered reads were subsequently de-replicated and de-noised using *dada2* default parameters to combine identical reads into amplicon sequence variants (ASVs) and construct consensus quality profiles for each combined set of sequences. The consensus quality profiles informed the de-noising algorithm, which infers error rates from samples and removes identified sequencing errors from the samples. Following the removal of chimeras, a consensus paired-end read file was generated for feature construction and downstream analysis. After feature table construction, taxonomic binning of classified sequences was built using a local copy of the Ribosomal Database Project (RDP) Classifier (Train Set 18, release 11.5),^103^ and normalized data were produced from the relative abundance of taxa present in each sample. A feature table of 13 458 unique ASVs with an average read length of 402 nucleotides in 198 samples was consequently constructed (after pre-processing, the minimum number of reads per sample was 26 623, and the average number of reads per sample was 46 768).

### Statistical analyses

Sequencing data were analyzed using bioinformatics and statistical analysis packages in R,^101^ including the packages *dada2* (version 1.12.1 ^102^), *vegan* (version 2.5.6), ^104^ *phyloseq* (version 1.28.0), ^105^ *ggplot2* (version 3.3.2), ^106^ and *CoDaSeq* (version 0.99.4) .^107,108^ For clinical and demographic data analysis, continuous variables were summarized as means (M) and standard deviations (SD) if normally distributed, or as medians (mdn) and interquartile ranges (IQRs) if non-normally distributed. Student’s *t*-tests and Mann-Whitney *U* tests were used to assess differences between normally and non-normally distributed data (normality tested using Shapiro-Wilk Normality Test), respectively. Categorical data were summarized as counts (*n*) and percentages, and *χ*^2^ or Fisher exact tests were used to assess differences between groups, where appropriate (for categories with only a few counts, Fisher exact tests were used). Significance was defined as *p* < 0.05.

Simpson’s index (using the *estimate_richness* function from the *phyloseq* package ^105^ in R), was used to evaluate α-diversity, as this measure is best suited for compositional data.^109^ Differences in α-diversity between different groups were evaluated using Wilcoxon rank-sum tests. Thereafter, taxa were agglomerated to species- genus- and phylum-level and abundance matrices were centered log-ratio (clr)-transformed (using *codaSeq.clr* in the *CoDaSeq* package), ^107^ using the minimum proportional abundance detected for each taxon for the imputation of zeros. The ordination of community variation was visualized using multidimensional scaling (MDS) of genus-level Aitchison distances (a beta diversity measure that evaluates sample dissimilarity and quantifies differences in the overall taxonomic composition between groups). The *capscale* function (from the *vegan* package, which performs a permutational ANOVA) was used to determine the contribution of metadata variables to microbiome community variation.^104^ To test whether a group of variables affected the ordination, the *ordiR2step* function (from the *vegan* package, which performs a forward stepwise model selection using permutation tests) was used. Statistical significance was defined as a false discovery rate- (FDR) corrected *q* ≤ 0.1, following correction for multiple testing (Benjamini–Hochberg procedure).

The ASV table was filtered to retain taxa that were observed in at least 15% of participants (to eliminate taxa with very low abundance/prevalence). This was followed by an exploratory approach, where variables of interest were tested for possible associations with relative taxonomic abundance on genus- and phylum-level. Associations between microbial composition data and categorical variables were analyzed with Wilcoxon rank-sum tests, while associations with continuous variables were tested using Spearman’s non-parametric correlation tests. The Benjamini–Hochberg procedure was applied for false discovery rate (FDR) correction for multiple testing (for the multiple taxa tested during each association test), and significance was defined as *q* ≤ 0.1.

Variables that were significantly associated with microbial composition were further investigated by fitting GLMs on clr-transformed data, after partialling out the effect of main microbiome covariates previously identified in a large Flemish cohort ^23^ and as described in literature ^110^ (age, sex, BMI, previous diagnosis (based on self-report medical questionnaire) of IBD/IBS/CeD, and the BSS). For associations that remained significant, additional variables that had a significant effect on the microbial composition in this study (henceforth referred to as additional microbiome covariates), namely current use of prescription medication, a previous COVID-19 infection, a COVID-19 vaccination, and alcohol intake in the last 2 weeks, were also included as potential covariates in the GLM. Significance was defined as *p* < 0.05.

## Supplementary Material

Supplemental Material

## Data Availability

The data that support the findings of this study are available from the corresponding author, [S Malan-Müller], upon reasonable request.

## Data deposition

The sequencing data have been deposited with links to BioProject accession number PRJNA887947 in the NCBI BioProject database (https://www.ncbi.nlm.nih.gov/bioproject/).
